# Notch and its oncogenic activity in human malignancies

**DOI:** 10.1007/s10353-017-0491-z

**Published:** 2017-09-18

**Authors:** Marlena Brzozowa-Zasada, Adam Piecuch, Marek Michalski, Oliwia Segiet, Józef Kurek, Marzena Harabin-Słowińska, Romuald Wojnicz

**Affiliations:** 1 Department of Histology and Embryology, School of Medicine with the Division of Dentistry in Zabrze, Medical University of Silesia, Jordana 19, 41-808 Zabrze, Poland; 2 Municipal Hospital, Jaworzno, Poland

**Keywords:** Notch, Cancer, Chemoresistance, Epithelial-to-mesenchymal transition

## Abstract

**Background:**

Increasing evidence has demonstrated that Notch signaling is deregulated in human hematological malignancies and solid tumors. This signaling has a protumorigenic effect but may also act as a tumor suppressor. How induction of a single pathway gives rise to the opposite effects in different cell types is still unknown.

**Methods:**

This review article includes available data from peer-reviewed publications associated with the role of Notch signaling during cancer pathogenesis.

**Results:**

Numerous reports have indicated that alterations in Notch signaling and its oncogenic activity were originally associated with the pathogenesis of T‑cell acute lymphoblastic leukemia/lymphoma (T-ALL), an aggressive hematologic tumor affecting children and adolescents. The possibility that Notch could play a significant role in human breast cancer development comes from studies on mouse mammary tumor virus-induced cancer. Numerous findings over the past several years have indicated that alterations in Notch signaling are also responsible for ovarian cancer development. Mention should also be made of the connection between expression of Notch 3 and increased resistance to chemotherapy, which remains a major obstacle to successful treatment. Notch as an oncogenic factor is also involved in the development of colon cancer, lung carcinoma and Kaposi’s sarcoma.

**Conclusion:**

Notch is a binary cell fate determinant and its overexpression has been described as oncogenic in a wide array of human malignancies. This finding led to interest in therapeutically targeting this pathway, especially by the use of gamma-secretase inhibitors (GSIs) blocking the cleavage of Notch receptors at the cell membrane by the inhibition of Notch intracellular domain (NICD) releasing. Preclinical cancer models have revealed that GSIs suppress the growth of cancers such as pancreatic, breast and lung cancer.

## Introduction

It is generally accepted that Notch signaling plays a fundamental role during embryonic development that is associated with the control of cell proliferation, differentiation and apoptosis. Moreover, a great number of studies have revealed that this pathway also shows a connection to postnatal processes. Noteworthy among these are hematopoiesis, mammary gland development, maturation of gastrointestinal epithelium, immune regulation, angiogenesis and neural stem cell survival [1, 2].

**Fig. 1:**
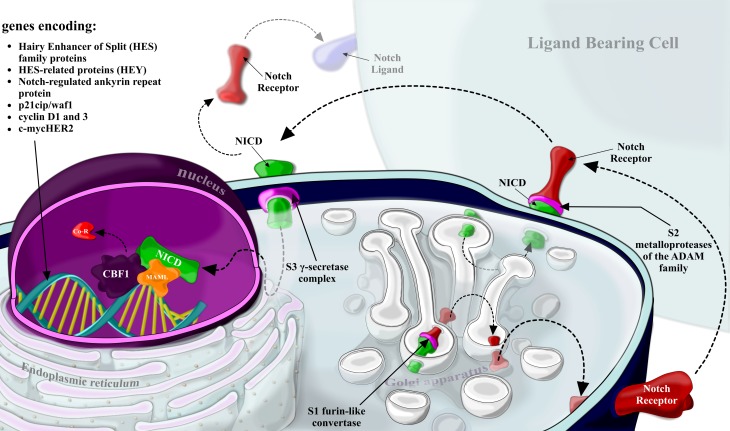
Notch receptors are synthesized as immature in the endoplasmic reticulum. Following proteolytic cleavage by furin-like convertase (S1 cleavage) in the trans-Golgi, mature Notch receptors accumulate on the cell surface as heterodimers composed of the Notch extracellular domain (*NECD*), the transmembrane domain (*NTM*) and the intracellular domain (*NICD*), held together by non-covalent interactions. Notch signaling-induced trans-activation is triggered by contact between a membrane-associated ligand on the signal-sending cell and a Notch-transmembrane receptor on the signal-receiving cell. The interaction with the ligand predisposes the Notch receptor to cleavage by ADAM metaloproteases (S2 cleavage), which allows subsequent cleavage by the gamma secretase (*GS*) complex (S3 cleavage). S3 cleavage leads to the release of active NICD from the membrane, which translocates to the nucleus and regulates the transcription of specific target genes including hairy enhancer of split (*HES*) family proteins, HES-related proteins (*HEY*), Notch-regulated ankyrin repeat protein and p21cip/waf1, cyclin D1 and 3, c‑myc and HER2

The Notch signaling pathway is composed of Notch receptors and Notch ligands. In humans the Notch receptors include Notch14. All receptors and their ligands, e. g. delta-like ligand (DLL1–4) and Jagged (JAG1–2), belong to the family of the single-pass transmembrane proteins characterized by multiple epidermal growth factor (EGF)-like repeats in the extracellular region. Notch receptors are synthesized as inactive single peptide precursors. These form before reaching the plasma membrane and are proteolytically cleaved by a furin-like convertases in the trans-Golgi network. The first cleavage (S1) generates non-covalently bound heterodimers that are composed of the *N*-terminal ligand-accessible Notch extracellular domain (NECD), and a C-terminal Notch transmembrane fragment (NTM). This fragment is composed of the extracellular stub, transmembrane domain and Notch intracellular domain (NICD) (Fig. 1).

The significant feature of the Notch pathway is juxtacrine acting between neighboring cells. During the first step of activation, Notch ligands bind to Notch receptors on an adjacent cell. During the second step Notch receptors undergo conformational changes followed by the second cleavage (S2), catalyzed by a member of a disintegrin and metaloprotease (ADAM) family (ADAM17 or ADAM10). The third cleavage (S3) is made by a presenilin-dependent γ‑secretase protease complex (an integral membrane protein complex) consisting of presenilin 1 (PSEN1or PSEN2, nicastrin, presinilin enhancer 2 [PEN2]) and anterior pharynx-defective 1 (APH1). After that, the active NICD is released into the cytoplasm and then to the nucleus, where it binds to the ubiquitous transcription factor CSL (CBF1/supressor of hairless, and longevity-assurance gene-1). NICD is known to convert a large co-repressor complex into a transcriptional activating complex. This complex is primarily composed of NICD, CSL, mastermind-like protein (MAML; a transcriptional coactivator), SKIP (ski-interacting protein as a CBF1 binding protein) and p300, and in this form induces the transcription of Notch target genes. Noteworthy among these are genes encoding hairy enhancer of split (HES) family proteins, HES-related proteins (HEY), Notch-regulated ankyrin repeat protein and p21cip/waf1, cyclin D1 and 3, c‑myc and HER2 ([3, 4]; Fig. 1).

Increasing evidence has demonstrated that Notch signaling is deregulated in human hematological malignancies and solid tumors [5]. This signaling has a protumorigenic effect, but may also act as a tumor suppressor [6]. How induction of a single pathway gives rise to the opposite effects in different cell types is still unknown. The Notch pathway probably turns on or turns off different target genes and downstream pathways [7]. This paper discusses the oncogenic activity of Notch in human malignancies.

## Notch signaling and T‑ALL pathogenesis

Numerous reports have indicated that alterations in the Notch pathway were originally associated with the pathogenesis of T‑cell acute lymphoblastic leukemia/lymphoma (T-ALL), an aggressive hematologic tumor affecting children and adolescents. Nevertheless, this disease also occurs in adults. Patients presenting with diffuse (>25%) bone marrow infiltration are diagnosed with T‑cell acute lymphoblastic leukemia, while those with mediastinal masses and limited bone marrow involvement are diagnosed as T‑cell acute lymphoblastic lymphoma [8].

The first evidence demonstrating that Notch might be the significant element in leukemogenesis came in the early 1990s when Ellisen and coworkers characterized a rare chromosomal translocation t(7;9)(q34;q34,3) involving the human Notch1 gene in T‑ALL [9]. However, the fundamental role of Notch1 in T‑ALL development was discovered in 2004 together with the discovery of activating Notch1 mutations in nearly 60% of TAALs. Weng et al. showed that Notch1 mutations occuring in the HD domain (20% of T‑ALL) resulted in a ligand-dependent induction of the receptor. In contrast, mutations in the PEST domain presenting in 15% of T‑ALLs were responsible for enhanced NICD1 stability and abnormally prolonged Notch1 activation [10]. Additionally, juxtamembrane expansion (JME) alleles and intragenic deletions encompassing the 5’ region of the Notch1 locus (Notch1 del-N) showed connections with overexpression of Notch1 in rare cases of T‑ALL [11, 12]. Several studies revealed that nearly 20% of T‑ALLs might be connected with mutations in the FBXW7 gene [13–15]. Those mutations have been linked to Notch1 PEST mutations and they led to increased ICN1 stability. It should be pointed out that FBXW7 mutations may also be related to another oncogenic activity, since this F‑box protein is involved in the degradation of oncoproteins such as MYC, JUN, MCL1 and cyclin E [16, 17].

Recent studies demonstrated a close dependency between the protumorigenic effect of Notch1 and the transcriptional regulation of cell growth and metabolism. Notch1 directly controls a great number of genes involved in anabolic pathways and also promotes cell growth through direct transcriptional upregulation of MYC [18]. The major results of Notch1 activation during the regulation of cell growth are further supported by the interaction of Notch1 signaling with the PI3K-AKT-mTOR pathway [19–21]. Notch1 transcriptionally upregulates molecules upstream of the PI3K such as the interleukin 7 receptor alpha chain (IL7RA), the pre-T-cell receptor alpha (PTCRA) and the insulin-like growth factor receptor (IGF1R) [22–24].

Among other significant mechanisms associated with T‑cell transformation downstream of oncogenic Notch, mention should be made of the promotion of the G1-S phase. Notch1 signaling is associated with the promotion of G1-S through upregulation of CDK4 and CDK6 and downregulation of p27/KIP1 and p18/INK4C cell cycle inhibitors [25–27]. Ntziachristos et al. demonstrated that Notch1 antagonized the activity of the Polycomb repressive complex 2 (PRC2) connected with writing the H3K27 mark. It is also worth noting that mutational loss of the PRC2 complex including EZH2, SUZI2, and EED has often been found in T‑ALL [28].

The question arises regarding the possible relation of Notch1 activation to clinical outcome in T‑ALL. Breit et al. showed that the presence of Notch1 mutations has been significantly correlated with prednisone response, favorable minimal residual disease (MRD) kinetics and improved long-term outcome in children with T‑ALL [29]. Analysis of the prognostic impact of both Notch1 and FBXW7 mutations in pediatric T‑ALL patients showed a favorable prognosis [30]. In contrast, Mansour et al. did not show any significant association between Notch1 and FBXw7 mutations and prognosis [31]. In this context, it is worth noting the study conducted by Ben Abdelali et al., who revealed that the favorable prognosis of Notch1/FBXW mutations in adult T‑ALL was found in more intense, pediatric-inspired GRAALL chemotherapy protocols but not in the less-intense LALA-94 study [32]. This could suggest that differences in therapy intensification may influence the prognostic impact of Notch activating mutations in T‑ALL.

It is worth noting here the identification of a novel chalcone derivative inhibiting Notch signaling in T‑ALL. By functional and biological analysis of the most representative molecules of an in-house library of natural products, Mori et al. designed and synthesized the chalcone-derivative 8 possessing the Notch inhibitory activity at concentrations between 5–10 µm. It has been demonstrated that this inhibitor suppressed cell growth of several human T‑ALL cell lines, including DND41, KOPTK1 and TALL-1. This suppression is connected with the activation of apoptosis and increased expression of p27. Moreover, G1 cell cycle arrest has also been detected. Importantly, compound 8 inhibited Notch signaling without interfering with S2 and S3 proteolytic cleavages, depending on ADAM and GS, respectively. Nevertheless, this requires further investigation and development [33].

Extensive research over the past half a century indicates that Notch also plays a very significant role in the pathogenesis of B‑cell malignancies. Alterations in the Notch pathway have been observed in Hodgkin’s lymphoma, Burkitt’s lymphoma, B‑cell chronic lymphatic leukemia, diffuse large B‑cell lymphoma, primary effusion lymphomas related to Kaposi’s sarcoma herpes virus infection and multiple myeloma (MM) [34].

## Aberrant Notch signaling as a factor affecting breast cancer development

The possibility that Notch could play a significant role in human breast cancer development comes from studies on mouse mammary tumor virus-induced cancer. This study demonstrated that Notch4 showed an ability to transform mammary epithelium in vitro and in vivo. Moreover, an activated form of this receptor slowed ductal growth and perturbed lobular outgrowth prior to tumor formation [35].

The majority of breast cancers express estrogen, progesterone receptors (ER,PR), HER2 and/or ErbB-2 [36]. Zardawi and colleagues demonstrated that increased expression of Notch1 was an early event in both murine and human breast tumor development. Importantly, there was a connection between the high level of Notch1 and the HER-2 molecular subtype of breast cancer [37]. In this context, the study of Li et al., who found that Notch1 activity was not a characteristic feature of primary ductal breast carcinoma, appears to be very interesting. However, the high level of Notch1 has been significantly correlated with poorer differentiation of breast and prostate tumors [38]. Rizzo et al. reported that 80% of epithelial hyperplasias of usual type (HUTs), 67% of ductal carcinomas in situ (DCISs), 89% of invasive ductal carcinomas (IDCs) and 57% of invasive lobular carcinomas (ILCs) revealed high expression of Notch1, while expression in the non-pathological samples has been characterized as low or negative [39]. Mittal et al. also revealed the high level of Notch expression in IDC in comparison to normal tissues [40]. Bolos et al. demonstrated that ectopic expression of the NICD1 in the MCF-7 breast adenocarcinoma cell line has been associated with a reduction in the E‑cadherin level. Increased migratory and invasive abilities have also been detected in MDA-MB-231 cells. Notch1 activation in the mouse mammary gland was responsible for the formation of papillary tumors characterized by enhanced expression of Hes1 and Hey1, and delocalized E‑cadherin expression. Interestingly, the growth of subcutaneous xenografts generated with MCF-7 cells was boosted after NICD1 activation in a cell-autonomous manner. This may indicate that Notch1 is a key player in epithelial-to-mesenchymal transition (EMT) in breast cancer [41]. Shao et al. obtained similar results. These researches revealed that activation of JAG1 was also connected with EMT induction. Probably, this event was also related to upregulation of NICD1 [42]. It is worth noting that Notch1 played a vital role in regulation of EMT in a Slug-dependent manner. The luciferase reporter assay further demonstrated that Notch1 positively regulated Slug expression by enhancing Slug promoter activity. Notch1 might downregulate Slug to maintain the epithelial phenotype and inhibit the migration and invasion of breast cancer cells [42]. Knockdown of Notch1 significantly inhibited cell proliferation, migration and colony formation. In addition, downregulation of Notch1 caused cell cycle arrest in the G0/G1 phase, but promoted apoptosis under in vitro conditions. To further verify this mechanism, it was found that activation of Notch1 could upregulate the expression of cyclin D1, survivin and Bcl-xL. Therefore, knockdown of Notch1 arrested the cell cycle at G0/G1. This molecular event is probably cyclin D1-dependent and the inhibition of apoptotic cell death might be related to enhanced expression of survivin and Bcl-xL. Li et al. suggested that Notch-induced cell invasive growth might be associated with upregulation of MMP-9, MMP-2 and VEGF. Notch1 signaling might promote breast cancer cell invasion due to the activation of NF-κB and its downstream target genes including MMP-2, MMP-9 and VEGF [43].

The presence of JAG1 in primary lesions indicated a higher risk of metastatic relapse. JAG1 expression correlated with vascular invasion and lymph node involvement [44]. Dickson et al. also demonstrated that patients with high levels of JAG1 had a worse outcome in comparison to those with low levels of expression. This elevated expression of Notch ligands has been associated with the metastatic ability of breast cancer cells [45]. Moreover, it has been demonstrated that high expression of JAG1 shows a correlation with breast cancer metastasis [46, 47]. The analysis of the gene expression showed JAG1 to be one of 17 cytokine/ligand genes overexpressed in bone metastasis in comparison to liver, brain and lung metastasis, implying a potential organ-specific function for tumor-derived JAG1 in metastatic colonization of the bone [48]. Knockdown of JAG1 in breast cancer cells suppressed bone metastatic ability without impacting on the growth of cultured cells and primary mammary tumors. It was thought that this pro-metastatic function of JAG1 was mediated by two mechanisms: Firstly, by inducing osteoblasts to release IL-6, thereby affecting the growth of cancer cells; and secondly, by stimulating severe osteolysis, which is associated with space formation for metastasis [49].

## Notch receptors and resistance to chemotherapy in ovarian carcinoma

Aberrant activation of the Notch signaling pathway has been associated with high grade ovarian carcinoma and carcinogenesis. Moreover, it plays a crucial role in chemoresistance in ovarian cancer patients [50]. The high level of Notch3 accompanied by a positive correlation with the expression of JAG1 and JAG2 has been detected in serous ovarian carcinomas. In addition, overexpression of Notch3 has been significantly correlated with advanced stage, lymph node metastasis, chemoresistance and poor overall survival [51]. In recurrent ovarian carcinoma enhanced expression of nuclear Notch3 also showed a link ro poor clinical outcome [52, 53]. The connection between the expression of Notch3 and increased resistance to chemotherapy, which remains a major obstacle to successful treatment, should also be mentioned here. Ectopic expression of Notch3 following transduction with NICD3 in ovarian surface epithelium and low-grade serous ovarian cancer cells led to increased resistance to carboplatin in vitro. In contrast, shRNA-mediated knockdown of Notch3 in OVCAR3 cells resulted in higher sensitivity to carboplatin compared with OVCAR3 cells transduced with a non-specific control shRNA [52]. Gupta et al. demonstrated that activation of Notch3 in OVCA429 cells was responsible for a fibroblast-like morphology and induction of the smooth muscle α‑actin, Slug and Snail. Interestingly, decreased expression of E‑cadherin has also been observed. This may suggest that Notch3 activation generates EMT in OVCA429 cells. Moreover, Notch3 activation rendered OVCA429 cells more resistant to carboplatin-induced cytotoxicity and impaired carboplatin-induced apoptosis [54]. Interesting results have been obtained by Kang et al., who demonstrated that Notch alteration impaired migration and angiogenesis and enhanced apoptotic cell death in paclitaxel-resistant cancer cells. The treatment of cells with Notch3 siRNA and gamma-secretase inhibitors (GSIs) was associated with arresting cells in the G2/M. At the same time, enhanced expression of proapoptotic proteins has been revealed [55].

The role of Notch1 in ovarian cancer was first described by Hopfer et al., who evaluated Notch mRNA expression in ovarian adenocarcinoma, borderline tumors and adenomas. They revealed more abundant expression of JAG2 and DLL1 in adenocarcinomas compared with adenomas. Interestingly, the stable transfection of A2780 cells with the NICD1 enhanced cell proliferation and increased colony-formation ability [56]. The high level of Notch1 has also been revealed in ovarian cancer cell lines including OVCAR3, SKOV3 and CaOV3. siRNA downregulation of NICD1 in those cells resulted in inhibition of cell proliferation. Notch1 also seems to be a very interesting factor in the context of chemoresistance [57–60]. Dysregulation of miRNA alters a network of functional targets and signaling pathways resulting in acquired chemoresistance in human cancers. Liu and coworkers demonstrated that increased expression of miR-199b-5p sensitized ovarian cancer cells to cisplatin-induced cytotoxicity. Conversely, re-expression of miR 199b-5p and siRNA-mediated JAG1 knockdown or treatment with GSI attenuated enhanced cisplatin-mediated cell cytotoxicity. It seems, therefore, that the epigenetic silencing of miR-199b-5p is significantly associated with acquired chemoresistance in ovarian cancer cells through the activation of JAG1-Notch1 [61]. The study by Zhou et al. provides evidence that downregulation of miR-449a could enhance ovarian cancer cell proliferation and induce cisplatin resistance. Importantly, inhibition of Notch1 signaling by overexpression of miR-449a could sensitize chemoresistant ovarian cancer cells to cisplatin-induced cytotoxicity. It may suggest that the miR-449a/Notch1 axis plays a significant role in the development of ovarian cancer [62]. Liang et al. revealed the role of miR-433. They reported that its overexpression significantly inhibited the migration and invasion of cancer cells, but had no significant effect on cell proliferation. Notch1 was a direct target of miR-433 and its downregulation inhibited the invasion of cancer cells [63].

Results of Alniaimi et al. demonstrated that Notch2 seems to be a tumour suppressor in ovarian carcinogenesis. The high expression of Notch2 mRNA has been significantly associated with poor PFS for all ovarian cancer patients, especially in grade II ovarian cancer. Interestingly, in serous and endometrioid cancer patients high expression of this mRNA was not correlated to poor PFS and OS [64].

## Notch signaling and other solid tumors

### Colon cancer

As revealed by studies, Notch receptors are known to be highly oncogenic in the case of colon cancer. Zhang et al. demonstrated that overexpression of Notch1 promoted proliferation, colony formation, cell cycling and tumorsphere formation of colon cancer cells in vitro. Notch1 was also responsible for the development and increased growth of implanted colon cancers in vivo [65]. In humans, expression of Notch1 has been positively associated with depth of invasion, lymph node metastases and tumor-node-metastasis (TNM) stage [66]. This is probably due to the role of Notch1 in EMT induction [67]. Fender et al. demonstrated that retroviral transduction of constitutively active ICN1 into HCT-116 cells resulted in increased expression of EMT and stemness associated proteins including CD44, Slug and Smad-3 [68]. Moreover, disease outcome depends on the crosstalk with other signaling pathways. For example, Gopalakrishnan et al. emphasized the strong interaction of NICD with NFκBp65. Importantly, Notch1 and NFκB could independently contribute to tumor progression, but their interaction is thought to be a determinant that would affect the clinical outcome of the disease and therapeutic intervention [69]. Kim et al. found that Notch1 is able to suppress the expression of Wnt target genes even when β‑catenin destruction by the adenomatous polysposis coli (APC) complex has been disrupted. Induction of Notch1 transformed high-grade adenoma into low-grade adenoma in an APC^Min^ mouse model of colon cancer and suppressed the expression of Wnt target genes through epigenetic modification connected with recruitment of histone metylotransferase SET domain bifurcated 1 (SETDB1) [70]. Microarray analysis revealed a negative correlation between the Notch1 target gene, Notch-regulated ankyrin repeat protein 1 (NRARP1) and Wnt target genes [70].

Notch2 expression has been reported to show a low level of expression in colon cancer. Expression of this receptor has been clearly correlated with tumor differentiation. Several studies demonstrated the negative correlation between Notch1 and Notch2. Increased expression of Notch1 or decreased expression of Notch2 is thought to be a risk factor for poor clinical outcome in colon patients [71, 72].

In contrast, Notch3 has been shown to be highly expressed in colon cancer. Nuclear expression of Notch3 has been correlated with tumor recurrence and may serve as a novel predictive marker in recurrent stage II and III colorectal cancer [73]. Notch3 signaling also induced the expression of Cdc42- and Rac1-specific guanine nucleotide exchange factor Asef in cancer cells. It is generally accepted that expression of this factor is stimulated by the tumor suppressor APC. Furukawa et al. revealed that mutant APC and Notch3 might cooperate to cause Asef-mediated aberrant colorectal tumour cell migration and adenoma formation [74]. Interestingly, results from this study also demonstrated that endothelial DLL-4 had the potential to induce colorectal cancer cell migration via Notch3 and Asef. DLL4 is known to be highly expressed in cancer-associated endothelial cells in colorectal cancer. It may indicate that this ligand modulates Notch activity by heterotypic cell interactions [75]. In colon cancer forced expression of Notch3 increased the level of MUSASHI-1 (MSI-1), whereas silencing of Notch3 by shRNA reduced the MSI-1 level in both colorectal cancer cells and tumor xenografts. Moreover, enforced Notch3 expression or stimulation by DLL4 enhanced levels of Notch1 in colorectal cell lines. What is important is that the treatment of cells with anti-Notch2/3 antibody increased expression of NUMB and significantly reduced the formation of tumor cell spheroids. This feed-forward circuit with DLL4, Notch3, MSI-1, NUMB and Notch1 may be relevant for the regulation of Notch during cancer formation [76].

In the context of colon cancer pathogenesis it is also worth noting microRNA. The expression patterns of these molecules help distinguish colon cancer from other diseases. Although it is known that miRNAs possess the ability to regulate gene expression by decreased translation or enhanced degradation of the target message, some studies demonstrated that Notch3 might be downregulated at both the protein and mRNA levels by miR-206 in mouse and humans. This suggests that miR-206 regulation is not restricted to the post-transcriptional level, but may also be detected at the transcriptional level [77]. The suppression of the Notch3 protein by miR-206 is probably possible by direct miR-206 binding to the 3’-UTR of Notch3. It seems, therefore, that the tumour suppressive capacity of miR-206 can be explained by both direct Notch3 signaling inhibition and indirect cross-talk with other signaling pathways involving CDH2 and MMP-9 [78].

### Lung cancer

For the first time Notch and its oncogenic activity in lung cancer has been discovered in a tumor-associated translocation between chromosome 15 and 19. The aberrant activation of the Notch pathway is a very characteristic feature of none-small cell lung carcinoma (NSCLC) [79]. The high level of Notch1 has also been clearly associated with poor clinical outcome in patients with lung adenocarcinoma [80]. Induction of Notch signaling through either Notch1 upregulation or Numb downregulation has been characterized in 30% of primary human NSCLC [79]. Allen et al. revealed that in the mouse alveolar epithelium, acute activation of NICD1 resulted in a wave proliferation and the formation of extensive hyperplasia, but the bulk of the alveolar hyperplasia was cleared from the lung periphery [81].

In lung cancer cell lines, Notch3 was upregulated, which was associated with karyotipic abnormalities [82]. In NSCLC this receptor has been abundantly co-expressed with EGFR to promote lung carcinogenesis [83, 84]. Notch3 is known to have a crosstalk with the EGFR-mitogen-activated protein kinase pathways leading to inhibition of apoptosis by BIM activity [85]. In the study by Yuan et al. a combined analysis of 19 eligible clinical studies demonstrated a predictive value for Notch1 and Notch3 expression in NSCLC patients. The results of the meta-analysis suggested that Notch1 is characterized as highly expressed in lung cancer compared with non-pathological tissue and is associated with lymph node metastasis and TNM stage. Importantly, expression of Notch3 has been related to lymph node metastasis. This may suggest that overexpression of Notch1 and Notch3 might have a prognostic value for overall survival [86]. In NSCLC patients DLL4 in cooperation with HES1 have also been positively related to poor overall survival [86].

### Kaposi’s sarcoma

In the context of Notch oncogenic activity, it is also worth mentioning Kaposi’s sarcoma (KS), which is a common neoplasm in HIV-1 infected individuals. Infection by KS-associated herpesvirus (KSHV) is a key factor in the development of KS [87, 88]. Interestingly, one of the mechanisms indicating the oncogenic effect of KSHV has been strongly associated with Notch activation. It was revealed that the replication and transcription activator (RTA) encoded by ORF50 induced its downstream genes and generated viral lytic reactivation by interplay with RBP-Jκ (CSL) which is known to be a crucial downstream effector of Notch [89]. It may indicate that RTA can take over the ability of Notch and mimic the function of NICD to modulate gene expression. On the other hand, Notch activation may react with the RTA promoter to reactivate KSHV from latency [90]. Some studies revealed that NICD is highly expressed in KSHV latently infected pleural effusion lymphoma (PEL) cells. Curry et al. also detected increased expression of Notch receptors, as well as the downstream target genes Hey1 and Hes1 in KS tumor cells under in vivo and in vitro conditions in comparison to endothelial cells, the precursor of KS cells [91]. The inhibition of Notch signaling by GSI in primary and immortalized KS cells has been connected with the activation of apoptosis in vitro. Moreover, injection of GSI into xenografted KS tumors on mice was related to tumour growth inhibition and tumor regression. Results of this study indicate that KS cells showed a high level of Notch expression and alterations in the Notch pathway inhibited KS cell growth [91]. Therefore, targeting Notch may be a very significant approach in KS patients.

In summary, Notch is a binary cell fate determinant, and its overexpression has been described as oncogenic in a wide array of human malignancies (Table 1). This finding led to interest in therapeutically targeting this pathway, especially by the use of GSIs blocking the cleavage of Notch receptors at the cell membrane by the inhibition of NICD releasing. Preclinical cancer models have revealed that GSIs suppress the growth of cancers such as pancreatic, breast and lung cancer. Nevertheless, GSI’s treatment in vivo is connected with significant side effects, especially in the gastrointestinal tract. Moreover, it must be noted that gamma-secretases influence the proteolytic cleavage of around 55 membrane proteins. Therefore, it is clear that GSIs are highly non-specific, and additional drugs must be designed that will target the Notch pathway more specifically [6].

**Table 1:** The expression of Notch receptors in human cancers

Type of cancer	Notch1	Notch2	Notch3	Notch4
T-ALL	Notch1 mutations in the HD domain resulted in a ligand-dependent induction of the receptor [10] Notch1 antagonized the activity of the Polycomb repressive complex 2 (PRC2) connected with writing the H3K27 mark. Mutational loss of the PRC2 complex has been often found in T‑ALL [28] The presence of Notch1 mutations has been significantly correlated with prednisone response, favorable minimal residual disease (MRD) kinetics and improved long-term outcome in children with T‑ALL [29]	–	–	–
Breast cancer	In all, 80% of epithelial hyperplasias of usual type (HUTs), 67% of ductal carcinomas in situ (DCISs), 89% of invasive ductal carcinomas (IDCs) and 57% of invasive lobular carcinomas (ILCs) revealed high expression of Notch1 [39] Ectopic expression of the NICD1 in MCF-7 breast adenocarcinoma cell line has been associated with the reduction of E‑cadherin levels [41] In MDA-MB-231 cells an increased migratory and invasive ability has also been detected [41] Downregulation of Notch1 caused cell cycle arrest in the G0/G1 phase but promoted apoptosis under in vitro conditions [43] Notch1 signaling might promote breast cancer cell invasion due to the activation of NF-κB and its downstream target genes including MMP-2, MMP-9 and VEGF [43]	–	–	Notch4 showed an ability to transform mammary epithelium in vitro and in vivo. Moreover, an activated form of this receptor slowed ductal growth and perturbed lobular outgrowth prior to tumor formation
Ovarian cancer	The active form of Notch1 (NICD1) was detected in ovarian cancer cell lines, ovarian cancer specimens and may lead to growth inhibition of ovarian cancer cells upon depletion of Notch1 by Notch1 siRNA [64] Downregulation of Notch1 inhibits cells growth, induces G1 cell cycle arrest and induces cell apoptosis in A2780 ovarian cancer cells High expression of Notch1 mRNA was not correlated to PFS for all ovarian cancer patients [64]	Notch2 seems to be a tumour suppressor in ovarian carcinogenesis [64] The high expression of Notch2 mRNA has been significantly associated with poor PFS for all ovarian cancer patients, especially in grade II ovarian cancer [64] Interestingly, in serous and endometrioid cancer patients high expression of this mRNA was not correlated to poor PFS and OS [64]	The high level of Notch3 accompanied by a positive correlation with the expression of JAG1 and JAG2 has been detected in serous ovarian carcinomas [51] Overexpression of Notch3 has been significantly correlated with advanced stage, lymph node metastasis, chemoresistance and poor OS [51–53] Ectopic expression of Notch3 following transduction with NICD3 in ovarian surface epithelium and low-grade serous ovarian cancer cells led to increased resistance to carboplatin in vitro [52] Activation of Notch3 in OVCA429 cells was responsible for a fibroblast-like morphology and induction of the smooth muscle α‑actin, Slug and Snail [54] The treatment of cells with Notch3 siRNA and gamma- secretase inhibitors (GSIs) was associated with arresting cells in the G2/M [55]	High expression of Notch4 mRNA was not correlated with PFS in all ovarian cancer patients; however, it was correlated to favorable OS [64]
Colon cancer	Overexpression of Notch1 promoted proliferation, colony formation, cell cycling and tumorsphere formation of colon cancer cells in vitro [65] The expression of Notch1 has been positively associated with depth of invasion, lymph node metastases and tumor-node-metastasis (TNM) stage [66, 67] Notch1 can suppress the expression of WNT target genes even when β‑catenin destruction by the adenomatous polysposis coli (APC) complex has been disrupted [70]	Increased expression of Notch1 or decreased expression of Notch2 is thought to be a risk factor for poor clinical outcome in colon patients [71, 72]	Nuclear expression of Notch3 has been correlated with tumor recurrence and may serve as a novel predictive marker in recurrent stage II and III colorectal cancer [73] Notch3 signaling also induced the expression of Cdc42- and Rac1-specific guanine nucleotide exchange factor Asef in cancer cells [74] The forced expression of Notch3 increased the level of MUSASHI-1 (MSI-1), whereas silencing of Notch3 by shRNA reduced the MSI-1 level in both colorectal cancer cells and tumor xenografts [76] The tumour suppressive capacity of miR-206 can be explained by both direct Notch3 signaling inhibition and indirect cross-talk with other signaling pathways involving CDH2 and MMP-9 [77, 78]	–
Lung cancer	Induction of Notch signaling through Notch1 upregulation has been characterized in 30% of primary human NSCLC [79] The results of the meta-analysis suggested that Notch1 is characterized as highly expressed in lung cancer in comparison to non-pathological tissue and is associated with lymph node metastasis and TNM stage [86]	–	In NSCLC Notch3 is abundantly co-expressed with EGFR to promote lung carcinogenesis [83, 84] Notch3 is known to have crosstalk with the EGFR/mitogen-activated protein kinase pathways leading to inhibition of apoptosis by BIM activity [85] The expression of Notch3 has been related to lymph node metastasis. Overexpression of Notch1 and Notch3 might have a prognostic value for OS [85, 86]	–

Notch was originally characterized as an oncogene, but studies have also revealed that this pathway may have suppressive activities in some hematopoietic cells, skin and pancreatic epithelium, as well as in hepatocytes [92]. Nicolas et al. revealed for the first time that mice with Notch1 deficient epithelia increased and sustained expression of Gli2, which is a downstream component of Sonic hedgehog (Shh), causing the development of spontaneous basal cell carcinoma-like tumours over time [94]. Another Notch target gene that appears to contribute to tumours’ suppressive effects in the epidermis is β‑catenin, a regulator of the Wnt pathway. Some studies demonstrated that Notch4 has been found to be negatively regulated by Notch in mouse keratinocytes in skin through p21 WAF1/Cip1, a negative transcriptional regulator of Wnt4a expression [93–96].
